# The Impact of Preprocessing Methods for a Successful Prostate Cell Lines Discrimination Using Partial Least Squares Regression and Discriminant Analysis Based on Fourier Transform Infrared Imaging

**DOI:** 10.3390/cells10040953

**Published:** 2021-04-20

**Authors:** Danuta Liberda, Ewa Pięta, Katarzyna Pogoda, Natalia Piergies, Maciej Roman, Paulina Koziol, Tomasz P. Wrobel, Czeslawa Paluszkiewicz, Wojciech M. Kwiatek

**Affiliations:** 1 Institute of Nuclear Physics Polish Academy of Sciences, PL-31342 Krakow, Poland; danuta.liberda@uj.edu.pl (D.L.); ewa.pieta@ifj.edu.pl (E.P.); natalia.piergies@ifj.edu.pl (N.P.); maciej.roman@ifj.edu.pl (M.R.); paulina1.koziol@uj.edu.pl (P.K.); czeslawa.paluszkiewicz@ifj.edu.pl (C.P.); wojciech.kwiatek@ifj.edu.pl (W.M.K.); 2 Institute for Medicine and Engineering, University of Pennsylvania, Philadelphia, PA 19104, USA

**Keywords:** FT-IR spectroscopy, prostate cancer cells, preprocessing, PLS-DA, PLSR, EMSC

## Abstract

Fourier transform infrared spectroscopy (FT-IR) is widely used in the analysis of the chemical composition of biological materials and has the potential to reveal new aspects of the molecular basis of diseases, including different types of cancer. The potential of FT-IR in cancer research lies in its capability of monitoring the biochemical status of cells, which undergo malignant transformation and further examination of spectral features that differentiate normal and cancerous ones using proper mathematical approaches. Such examination can be performed with the use of chemometric tools, such as partial least squares discriminant analysis (PLS-DA) classification and partial least squares regression (PLSR), and proper application of preprocessing methods and their correct sequence is crucial for success. Here, we performed a comparison of several state-of-the-art methods commonly used in infrared biospectroscopy (denoising, baseline correction, and normalization) with the addition of methods not previously used in infrared biospectroscopy classification problems: Mie extinction extended multiplicative signal correction, Eiler’s smoothing, and probabilistic quotient normalization. We compared all of these approaches and their effect on the data structure, classification, and regression capability on experimental FT-IR spectra collected from five different prostate normal and cancerous cell lines. Additionally, we tested the influence of added spectral noise. Overall, we concluded that in the case of the data analyzed here, the biggest impact on data structure and performance of PLS-DA and PLSR was caused by the baseline correction; therefore, much attention should be given, especially to this step of data preprocessing.

## 1. Introduction

Prostate cancer is one of the most common and deadliest types of cancers among men in developed countries [1]. Currently, its incidence among men in the US is similar to that of breast cancer among women. Even though prostate cancer can grow very slowly and sometimes in an early stage does not significantly influence the quality of the life of some patients, its early diagnostics and monitoring are crucial for treatment, which otherwise will be ineffective against the advanced stage of the disease. In vitro cell cultures of isolated human cells are powerful model systems to study malignant transformation and to determine whether normal, primary, or metastatic cells differ in morphology, chemical structure, proliferation, motility, or response to anticancer drugs. By analyzing a panel of normal and transformed prostate cells, the evolution of such differences can be correlated with the disease state and thus, suggest more effective drug treatment or serve as an additional prognostic factor.

**Figure 1:**
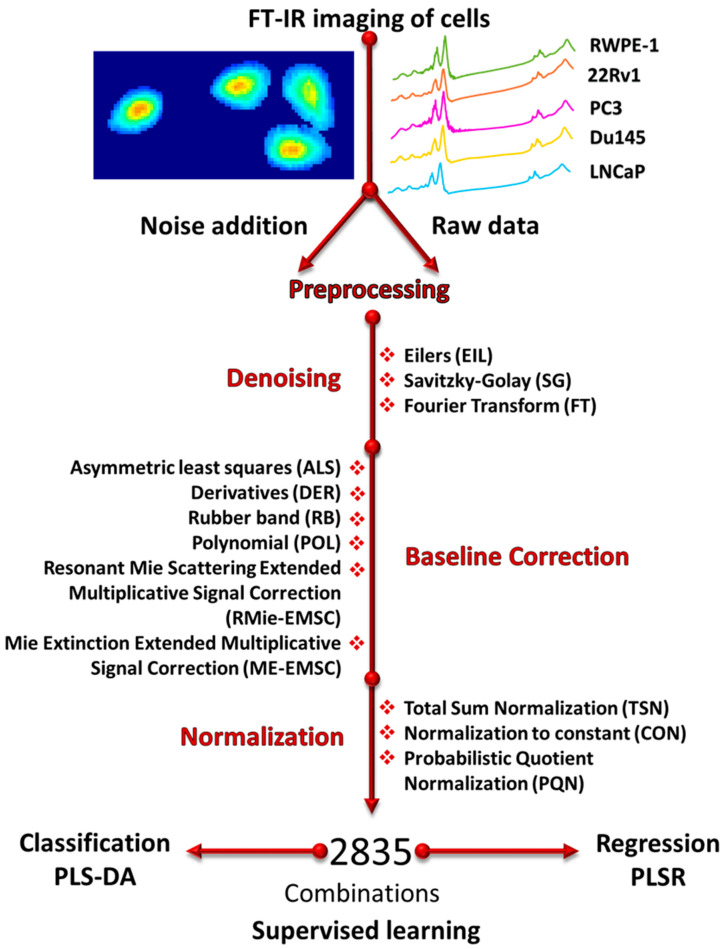
A scheme of the preprocessing steps—five prostate cell lines were imaged with FT-IR, then white noise was added to the original spectra. Raw data and data with added noise were preprocessed in the following order: denoising → baseline correction → normalization. Individual methods coming from one preprocessing step were combined with each method from the remaining two preprocessing steps. Taking into account the above and the number of parameters adjusted for each method, the number of combinations (data sets) was equal to 2835. All of these combinations were then used to create a classifier discriminating cell lines and a regression model of class assignments giving more detail about the relative importance of different preprocessing factors and parameters.

Spectroscopic methods are widely applied to study the evolution of biochemical compounds that can be subsequently elevated or diminished during malignant transformation in single cells [2,3,4,5]. Methods like Fourier transform infrared spectroscopy (FT-IR) and Raman spectroscopy are well established in biomedical applications [6,7,8,9,10,11,12,13] and offer a label-free biochemical snapshot of various biological processes. They have been successfully applied to prostate cell line discrimination in the past [14,15,16,17,18,19,20,21,22,23]. The biochemical sensitivity of IR spectroscopy usually needs to be leveraged by the application of advanced multivariate algorithms, which opens up a whole field of optimization of such approaches [24,25,26,27]. It is known that every specific data structure will change the optimal parameters of most optimizations; therefore, there is a need to perform optimization for a given problem [28,29,30,31,32,33,34,35,36,37,38]. Here, we present the discrimination of five prostate cell lines: one RWPE-1 cell line derived from the peripheral zone of the histologically normal adult human prostate and four cancerous cell lines derived from prostate cancer (22Rv1), and its metastasis to lymph nodes (LNCaP), the brain (Du145), and bone (PC3). We also perform a comparison of several state-of-the-art methods commonly used in FT-IR biospectroscopy (Figure 1). These include three steps of preprocessing: denoising, baseline correction, and normalization. In each step, we test known methods with the introduction of new ones. Baseline correction can be done in either a signal processing approach or using a physics-based model. Resonant Mie scattering extended multiplicative correction (RMie-EMSC) [39] has been the standard, but long computation time and the influence of reference spectrum on the corrected spectra shapes led to the development of a more accurate and stable upgrade in the form of Mie extinction extended multiplicative signal correction (ME-EMSC) [40], which has been recently released. RMie-EMSC and ME-EMSC were compared on the pancreas tissue spectra in our previous work [41]. Both algorithms gave similar results in terms of spectral correction, but RMie-EMSC was around 20 times slower than ME-EMSC. In the denoising part of preprocessing, we have already compared most of the known methods for imaging purposes [28]. However, the method proposed by Eilers et al. [42], which is not commonly applied in IR spectroscopy, is a potentially efficient algorithm for single spectra. Finally, in the normalization part, we tested the probabilistic quotient normalization (PQN) method [43]. This method is more commonly used for the normalization of data acquired with nuclear magnetic resonance spectroscopy [44]. We compared all of these approaches in their effect on the data structure, classification, and regression capability on experimental data (Figure 1). Additionally, we explored the influence of added spectral noise to highlight the sensitivity to noise of different methods. Our results show that even for the same data, it is important to optimize the classification and regression approaches separately, as the optimal parameters are different.

## 2. Materials and Methods

### 2.1. Cell Culture

PC-3, LNCaP, Du145, and 22Rv1 cells were grown in RPMI-1640 with L-glutamine, and 25 mM HEPES (Corning, Mediatech Inc., Manassas, VA 20109, USA) supplemented with 10% FBS (ATCC 30-2020) and 1% pen/strep. RWPE-1 cells were grown in Keratinocyte-SFM (Gibco, Life Technologies) supplemented with 1% pen/strep.

### 2.2. Sample Preparation for Spectroscopic Studies

Cells were seeded onto CaF_2_ optical windows at a density of 40.000 cells/mL. 48 h after seeding, cells were washed for 15 min in HBSS supplemented with calcium chloride and magnesium chloride (Gibco, Invitrogen, #14025) at 37 °C and fixed in 4% PFA solution (Affymetrix, Cleveland, OH, USA) in PBS for 20 min at 37 °C. Cells were washed and dried in a gradient of HBSS from 100% to 0% including: 100%, 90%, 80%, 70%, 60%, 50%, 40%, 30%, 20%, 10%, and 0% (ddH2O) and air-dried. Each step was performed 1 time for 2 min. All samples were washed and dried at room temperature.

### 2.3. FT-IR Measurements

FT-IR data were collected using a HYPERION 3000 FT-IR microscope (Bruker Optics, Ettlingen, Germany) equipped with a 36× objective and liquid nitrogen cooled 64 × 64 Focal Plane Array (FPA) detector (1.1 µm projected pixel size). The FT-IR spectra of fixed cells seeded onto CaF_2_ (13 × 1 mm) were acquired in a transmission mode in the range from 3800 cm^−1^ to 900 cm^−1^. The spectral resolution was 4 cm^−1^ (with a zero-filling factor of 1, giving rise to 1582 spectral points), and the number of scans per spectrum was 256. Spectra from each of the cells were averaged to a single spectrum, giving rise to a set of 48 spectra across the 5 cell lines: RWPE-1 (14 spectra), 22Rv1 (7 spectra), PC3 (7 spectra), Du145 (10 spectra), and LNCaP (10 spectra). The above approach provides data with the dimensionality of 48 objects (cells) by 1582 variables (frequencies).

### 2.4. Noise Addition and Preprocessing

In order to understand the influence of spectral noise on the classification outcome, a simulated homoscedastic noise was added to the original spectra as previously reported [28] to achieve Signal to Noise Ratio (SNR) corresponding to only 32 scans per spectrum. Before baseline correction, spectral ranges related to carbon dioxide and water vapor were removed. Since some baseline correction methods require these removed regions to correct baseline properly, the carbon dioxide region was interpolated. Raw data and data with added noise after carbon dioxide removal can be seen in Figure S1 in the Supplementary Materials. Computations were done using *MATLAB* software with internal implemented algorithms (Savitzky–Golay, Fourier transform, PLS) and self-written scripts. The baseline correction algorithms based on extended multiplicative scattering correction: RMie-EMSC created by Bassan et al. [45] and ME-EMSC created by Solheim et al. [40], were used as supplemented by the authors. Reasonable parameter ranges, which after correction provide spectra of good quality, were chosen by an experienced spectroscopist based on knowledge of the optical properties of cells. The number of data sets for each method is equal to the number of values of adjusted parameters in chosen ranges. After the baseline correction step, the carbon dioxide spectral region was trimmed again. Before Principal component analysis, spectra were normalized to the maximum value in each dataset.

### 2.5. Regression and Classification

The dependent variable Y used in regression task was coded as follows (−2, −1, 0, 1, 2) in accordance with the assumed cancer progression and metastasis character: benign RWPE-1 cells, primary prostate cancer 22Rv1 cells, and metastatic cell lines in arbitrary order (PC3, Du145, LNCaP). For the classification task, a matrix of cells membership to a given class (codded as 0 if a cell did not belong and 1 if the cell did belong to a given class) with dimensionality 48 objects (cells) by 5 variables (classes) was created. Based on the prediction of 5 different two-class models for each of the classes, the object was assigned to a class with the highest predicted class membership.

### 2.6. Model Calibration and Validation

Creation of the model set used for calibration (internal validation) and independent test set used for prediction power assessment (external validation) was done with the Kennard and Stone algorithm [46]: 75% and 25% of samples were chosen independently from each class to the model and test sets, respectively. The above approach gave 34 and 14 spectra in model and test sets, respectively. Model and test sets preprocessing were done independently. Leave-one-out cross-validation (LOOCV) was used to estimate the root mean square of cross-validation (RMSECV) for overfitting diagnosis in partial least squares regression (PLSR)—internal validation. The most appropriate latent variable (LV) number was chosen based on RMSECV as follows: in the range of LVs between 1 and 8, the LV giving the lowest RMSECV was chosen, unless in this range local minimum followed by two LVs higher than this minimum appeared—in this case, the LV number giving this local minimum was taken for model creation. The most appropriate LV number in partial least squares discriminant analysis (PLS-DA) was chosen based on accuracy values coming from internal validation (LOOCV), as follows: in the range of LVs between 1 and 8, the LV giving the highest accuracy was taken for model creation. Test RMSEP (root mean square of prediction) for the independent test set (external validation) was calculated to obtain the model prediction power in PLSR. The assignment of the sample to a class in classification with PLS-DA was done with the maximum predicted value criterion; model prediction power was assessed with accuracy values.

### 2.7. Description of Methods

State-of-the-art preprocessing methods that are used in IR spectroscopy were compared.

#### 2.7.1. Baseline Correction

In the Savitzky–Golay second derivative (DER) method, two parameters must be optimized; the first is associated with frame width, and the second with a polynomial degree. A polynomial of a set degree is fitted to the frame with the proper width, and next, the second derivative of this local polynomial is calculated [47]. The parameters range selected for this work were polynomial degrees 2 and 3 and a window size of 19:29 points.

The rubber band (RB) baseline correction method requires optimization of intervals between variables/frequencies. Between *s* points defining intervals, the minimum point is found, and a straight line or spline is fitted [48]. Then it is subtracted from the original data. Nine frequencies for intervals were chosen using spectroscopic knowledge: 1001 cm^−1^, 1280 cm^−1^, 1302 cm^−1^, 1761 cm^−1^, 1977 cm^−1^, 2412 cm^−1^, 2825 cm^−1^, 2997 cm^−1^, and 3519 cm^−1^.

The polynomial fitting (POL) method is based on the fitting of a polynomial with a chosen degree to a set of frequencies given by the user. In the next step, this polynomial is subtracted from a spectrum. In our research, frequencies were the same as for the RB method. A polynomial of degree 3:5 was chosen as optimal.

The asymmetric least squares smoothing (ALS) method relies on Whittaker smoother baseline estimation and least squares deviations weighting. Optimization of two parameters needs to be performed: a smoothing parameter *λ* and parameter *p* applied for weights *w_i_* calculations (introduction of this parameter gives higher weights for negative residuals $\left(y_{i}-z_{i}\right)$ and lower weights for positive one). The above approach is expressed by the equation:

(1) $$S=\underset{i}{\sum }w_{i}{\left(y_{i}-z_{i}\right)}^{2}+\lambda \sum {\left(\Delta^{2}z_{i}\right)}^{2}$$

where: *w_i_*—weights (if *y_i_ > z_i_ w_i_* = *p*, if *y_i_ < = z_i_ w_i_* = *1−p*), *y_i_*—signal, *z_i_*—rough signal [49]. Parameters which were chosen after optimization: *p* = 0.1, *λ* = 10^6^:10^8^.

Resonant Mie scattering extended multiplicative signal correction (RMie-EMSC) is a physics-based model for generating Resonant Mie scattering curves is coupled with the extended multiplicative scattering correction to correct distorted infrared spectra [39,45]. For each experimental spectrum, the algorithm estimates the refractive index across all the spectral ranges based on experimental data and reference spectrum. This is done in an iterative manner where for each iteration, a multitude of RMie curves (for combinations of physical parameters) is generated and decomposed with principal component analysis to extract loadings matrix. It is used to perform correction and model reference spectrum for potential further iterations. As a starting point, it can use either a reference Matrigel spectrum or an average of the experimental signal being corrected. The parameters to optimize are the choice of reference spectrum and the number of iterations. Reference Matrigel spectrum and 5 iterations were found to be sufficient the remaining physical parameters were set as recommended by Bassan et al. in Resonant Mie Scattering (RMieS) EMSC correction guide [50].

Mie extinction extended multiplicative signal correction (ME-EMSC) is the most recent improvement of RMie-EMSC in the form of open-source code [40], implementing a few features [51] in terms of the way certain estimations are made, which was already described in our recent publication [41]. It results in a faster and more stable algorithm with a ‘PreRun’ function for optimization of parameters. In this study, the Matrigel spectrum and default parameters were set as optimal for baseline correction except for the lower and upper ranges for scattering particle diameter, which were set to 2 and 8, respectively, to preserve the same input parameters as for RMie-EMSC.

#### 2.7.2. Normalization

Normalization to constant (CON) is one of the most frequently used methods of normalization, where normalization is performed to the most stable band/variable [52]. In the case of the data analyzed here, the Amide I band (1656 cm^−1^) was the most stable and was chosen for normalization.

In the total sum normalization (TSN) method, each spectrum is divided by the sum of its absorbances [52]. This method introduces an artificial correlation between variables because it is dedicated to closer data normalization.

In the first step of probabilistic quotient normalization (PQN), a standard—mean spectrum ($x_{s}$)—is calculated, then the ratios of corresponding features of the spectrum ($x_{i}$) and standard are computed. In the second step, a scaling factor—median of the ratios—is calculated. In the third step, spectra are divided by a corresponding scaling factor. This method has one assumption that the majority of the individual spectra and standard ratios are stable [43].

(2) $$x_{n}=\frac{x_{i}}{median\left(x_{i}/x_{s}\right)}$$

#### 2.7.3. Denoising

The principle of the Eilers (EIL) method is the same as in the ALS baseline correction method, with the difference that now the signal is estimated, rather than the baseline, and therefore weights are equal to one [42]. The optimal smoothing parameter λ for analyzed spectra was found to be 2:24.

In the Savitzky–Golay (SG) method, denoising is done by fitting the polynomial to a set of points indicated by frame width. The fitting process is executed multiple times until the moving window covers all spectral variables. The polynomial used in this method was optimized to be a degree of 2:3, frame size of 11:29.

Fourier transform (FT) is based on the transition from the signal domain to the frequency domain. Spectra are fitted by a series of sine and cosine functions, which after summation, reconstruct the original signal [53]. The optimization is done by adjusting the threshold used to reject high-frequency components related to noise. In our research, the threshold—size of the window in which low frequencies are included was set at 100:320 points.

## 3. Results and Discussion

### 3.1. Spectral Changes

**Figure 2:**
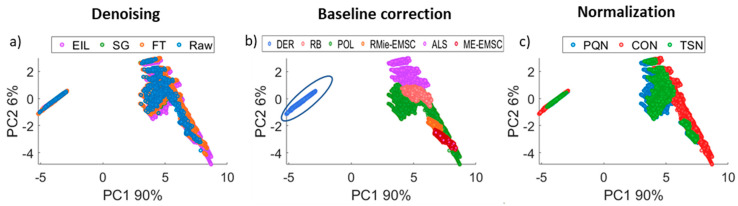
Principal component analysis exploration of the original data structure after application of three preprocessing steps. Each point corresponds to the individual spectrum coming from the original dataset on which unique combinations of the three steps were used. Subsections a, b, and c present the same PC projection but are colored according to a single preprocessing type: (**a**) denoising, (**b**) baseline correction, and (**c**) normalization. For better understanding, a set of spectra on which combinations of DER baseline correction method with other preprocessing steps are marked with a circle.

In the first step, a comparison of the relative effect of preprocessing steps on the spectral shapes was performed using principal component analysis (PCA). The first two PCs covering 96% of variance are plotted in Figure 2.

According to PCA, the three preprocessing steps can be ranked in their relative influence on the spectra, i.e., baseline correction > normalization > denoising. Figure 2a presents the impact of the denoising step on the data. Combinations for all denoising methods were in the same PC1 and PC2 space, which indicates that preprocessing step had no significant influence on the shape of the spectra. However, this was a high SNR dataset since 256 scans were co-added, and spectra averaging was performed. In order to observe the effect of noise, we simulated a noisier dataset by incorporating noise levels corresponding to 32 scans (based on our previous work [27]) into the averaged spectra. Noise addition has very little influence on PCA preprocessing steps ranking, as can be seen in Figure S2 in the Supplementary Materials. Exploration of baseline correction methods (Figure 2b) clearly shows the separation of the Derivative (DER) baseline correction method from the others in PC1 (90%). This deviation is caused by a significant change in the shape of spectra and is an expected and desired feature of derivatives. The next two significantly outlying methods are the resonant Mie extended multiplicative signal correction (RMie-EMSC) and Mie extinction extended multiplicative signal correction (ME-EMSC), and they can be distinguished from the remaining baseline correction methods in the PC2 (7%). This result was less expected, but RMie-EMSC and ME-EMSC are physics-based models and have more assumptions about the underlying data structure and, therefore, altered them in a more significant matter. The widest point spread was observed for the polynomial (POL) method in PC2 (7%)—it points out that the shape of spectra after POL baseline correction was highly altered. Asymmetric least squares (ALS) and rubber band (RB) partially overlapped, but ALS correction also gave spectra with shapes different than other baseline correction methods. Figure 2c shows the influence of the normalization preprocessing step on the data. It could be observed that probabilistic quotient normalization (PQN) and normalization to constant (CON) differed the most from each other. Total sum normalization was somewhat in between but it was more similar to PQN. POL combined with CON also gave the most different shape of spectra—points were located in space free from other combinations.

### 3.2. PLS Discriminant Analysis

**Figure 3:**
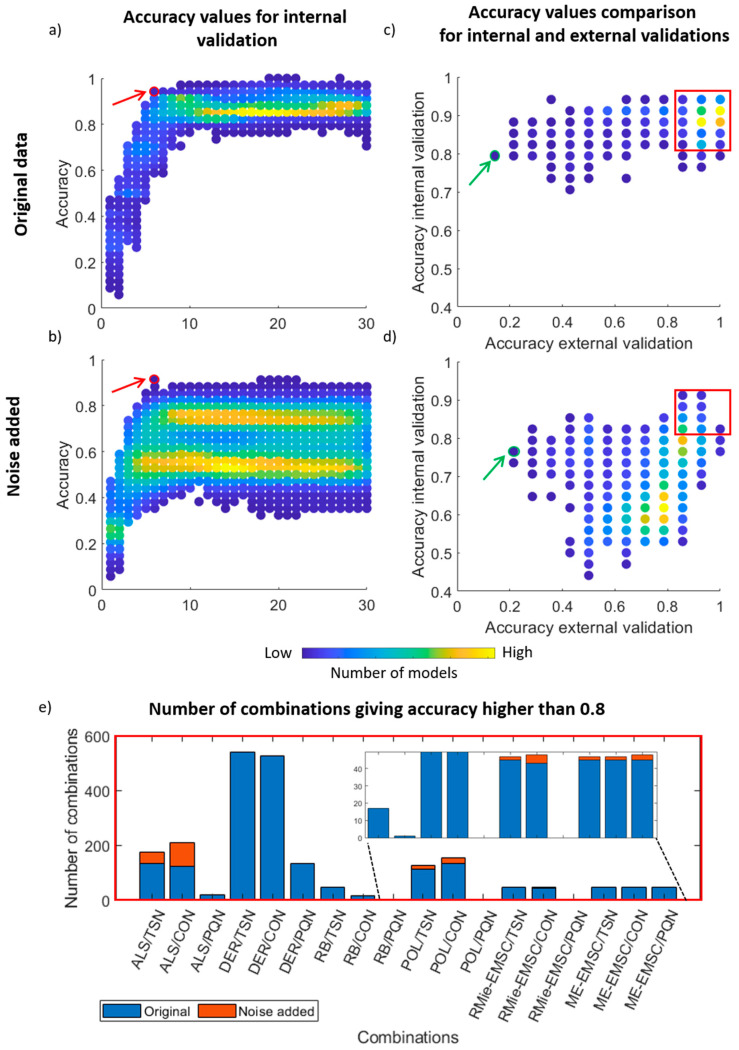
Results of PLS-DA classification: Values of accuracy for each combination of the methods (marked with dots, with yellow corresponding to a high number of models while blue to low number) calculated for up to 30 LVs of: (**a**) original data and (**b**) data with added noise. The red circle indicates the best combination of methods which gave very high internal accuracy with the smallest reasonable LVs number. Comparison of internal and external validation accuracy values (for the best LVs chosen based on internal validation) for (**c**) original data and (**d**) data with added noise. The green circle indicates the worst combination of methods which gave very high internal validation and low external validation accuracy values. (**e**) Number of combinations giving accuracy higher than 0.8 for internal and external validation—marked with a red frame on right figure panel: for original and noise added data divided into baseline/normalization categories.

Baseline correction was found to have the highest influence on the spectral shapes, but this does not necessarily mean the highest influence on the classification capability of PLS-DA. The information critical for classification may be distributed elsewhere and might not be affected by this correction. In order to investigate the relative influence of the preprocessing steps, a PLS-DA was performed (Figure 3) on the original, high SNR dataset (Figure 3a,c), and on a noisy dataset (Figure 3b,d). Since PLS results depend heavily on the chosen number of latent variables (LVs), the accuracy of the classifier was plotted against the number of LVs (from 1 to 30 for model creation).

For the original data (Figure 3a), most of the combinations were placed between 10 and 30 LVs with an accuracy higher than 0.8. However, LVs in this interval can be risky because more unwanted information (water vapor interference) or noise can be used by the model and make it less robust. The best combinations of methods that gave very high internal accuracy with the smallest reasonable LVs number (marked with the red circle in Figure 3a) contains 12 combinations listed in Table 1. The FT method combined with DER and CON/TSN normalization method is one of the most frequent combinations, which gave high internal accuracy for LV 6. Beta coefficients (colored the same as cell classes) for one of the best combinations (which gives high external validation accuracy equal to 0.86) marked with green color in Table 1 are shown in the Supplementary Materials in Figure S3a. The differences were clearly visible for proteins Amide I and Amide II (1500–1700 cm^−1^), Amide A and Amide B (3000–3400 cm^−1^); nucleic acids (944–1140 cm^−1^) and CH_2_/CH_3_ (2800–3000 cm^−1^) spectral regions—in-depth discussion of this can be found elsewhere [23]. Following this, accuracy values for external and internal validation for chosen LV obtained for each combination were compared. The high number of methods gives accuracy values for external and internal validation above 0.8. However, some methods result in internal accuracy above 0.8, but they gave a poor prediction for the independent test set—below 0.2. The model was overfitted, which resulted from a small number of samples, i.e., PQN method use mean spectrum as standard; therefore, this method is fragile for spectral distortion, which can occur after the first preprocessing step—baseline correction. Assuming that the test set contains a small number of samples, if certain spectra are being distorted, the majority of the individual spectra and standard ratios can be unstable. The method which gives the worst external accuracy and the best internal accuracy values for original data is a combination of FT/POL and PQN (marked with a green circle in Figure 3c). Spectra and beta coefficients for this combination are shown in Supplementary materials (Figure S4a). Denoising methods had no significant influence on classification stability for this dataset. However, data with added noise showed that classification performance was worse than for original data (Figure 3b,d), as was expected. Now three clusters of method combinations could be observed. In Figure 3b, most of the combinations were placed between accuracy equals 0.4–0.85, whereas the original data had accuracy intervals of 0.8–0.94. In noisy data case, there were 13 combinations which gave the best accuracy value with a reasonable number of LV equal to six (marked with a circle in the Figure 3b and listed in Table 1), consisting of SG denoising (the most frequent method), EIL, and FT, with the ALS baseline correction method and the CON normalization method. One of the combinations with the highest internal and external accuracy values (marked with green color in Table 1) is presented in Supplementary Materials (Figure S3b). Beta coefficients for data with added noise are different with the same spectral regions as for original data (Figure S3b in Supplementary Materials). Comparison of accuracy values for external and internal validation (Figure 3d) shows that there were two clusters of combinations, but accuracy was below 0.8 for both validations. In comparison to the original data (Figure 3c), the smaller number of methods provided external and internal accuracy values higher than 0.8. The most overfitted model was the combination of FT/RB and PQN (presented in Supplementary materials Figure S4b). Furthermore, the number of combinations in which accuracy was above 0.8 for external and internal validation was inspected (Figure 3e) to find which combinations were the most robust. Baseline correction methods had a crucial impact on accuracy values. In the case of original data, the DER method was found to be the most stable and achieved accuracy above 0.8 most frequently. ALS method is the most robust baseline correction method for noise-added data sets. This change in the best-performing baseline correction method was due to the fact that each derivation of signal adds a certain amount of noise. For high SNR, this was acceptable; however, if the starting SNR is not that great, the derivation of added noise lowers the capability of the models. Normalization methods had a smaller impact on PLS-DA classification. The most stable were combinations with CON and TSN and, while PQN normalization was the most unstable.

A general comparison between internal and external accuracy values for original and noise added data (Table 1) reveals that these values for noise added data are much closer to each other than for raw data. Noise added to raw data can mask artifacts like water vapor present in the spectra and other spectral distortion.

**Figure 4:**
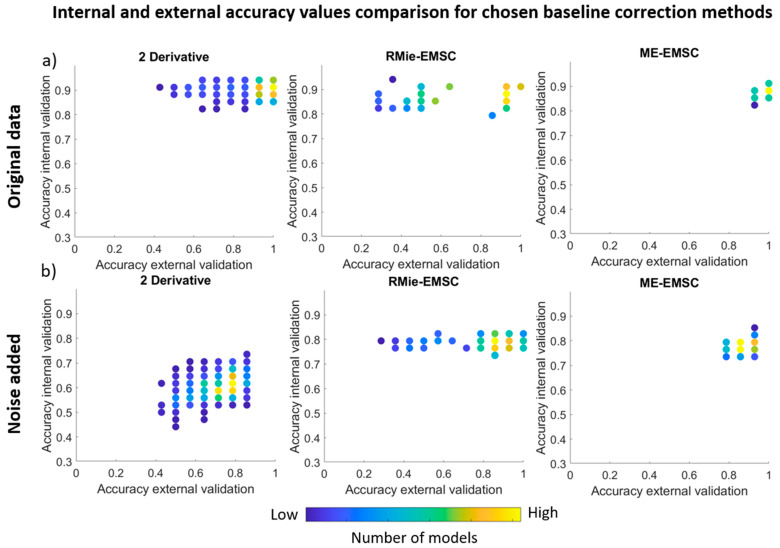
Internal and external accuracy values comparison for the best LVs for: (**a**) original data and (**b**) noise added data, for baseline correction methods: DER, RMie-EMSC, and ME-EMSC.

As we presented above, baseline correction methods have a crucial impact on classification results. Therefore, in Figure 4, we decided to compare three interesting cases of classification results for baseline correction methods. DER gives very high accuracy values for external and internal validation in the case of original data; however, for noise added data, accuracy values were moved in the direction of lower values. For original and noise added data ME-EMSC seemed to be the most stable and gave high internal and external accuracy values for both original and noise added data sets, while RMie-EMSC gave accuracy values that were spread along the external accuracy validation *x*-axis. Therefore, ME-EMSC was more stable in comparison to RMie-EMSC. Results for DER were also spread along the x-axis, but as it was related to the high number of adjusted parameters—some of the adjusted parameters could give higher data distortion and worse classification results. This comparison for the remaining baseline correction methods can be seen in the Supplementary Materials (Figure S5).

### 3.3. PLS Regression

**Figure 5:**
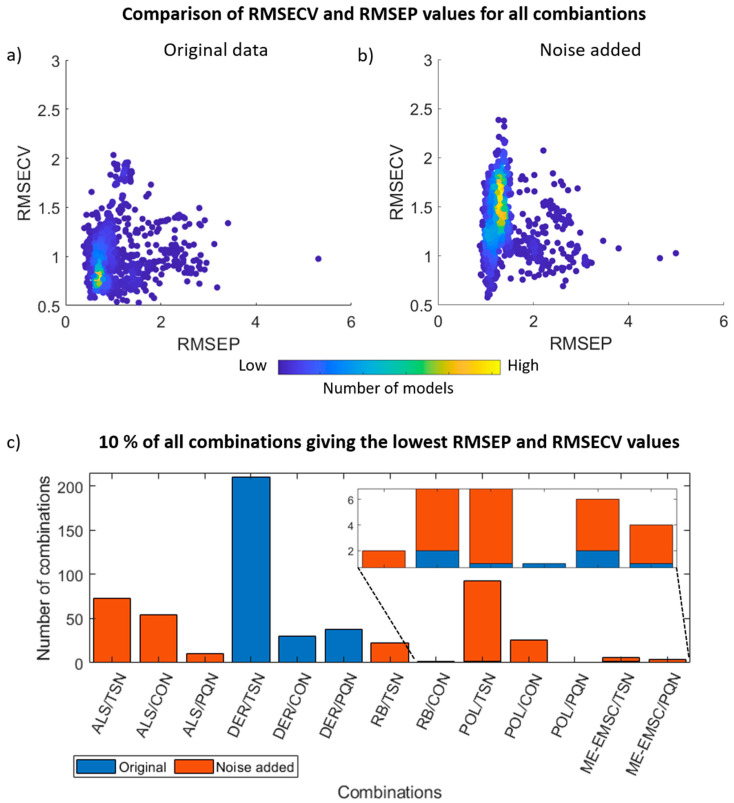
Comparison of RMSECV and RMSEP values for: (**a**) original data and (**b**) noise added data. Each dot on the plot presents a value for one combination of preprocessing methods. (**c**) Histogram of 10% of all combinations giving the lowest RMSECV and RSEMP for the original and noise added data.

Classification is often the most desirable outcome of an IR experiment, but in many situations, a regression to an independent variable is of interest. Moreover, very good classification (as in this example) can be reached with a large number of combinations, and care must be taken that the discrimination is not based on spectral artifacts amplified by a given preprocessing method. A better insight into model consistency is offered by PLSR. In Figure 5a,b, relations between model error values of internal validation (RMSECV) and external validation (RMSEP) for original and noise added data, respectively, are presented. In the case of original data, most of the combinations were placed close to 0.5; in the case of noise added data, some methods give values close to 0.5, but the center of gravity values were moved between 1.5 and 2 for internal validation and 1 for external validation. Relations between RMSECV and RMSEP for baseline correction methods are presented in Figure S6 in Supplementary Materials. The robustness of methods was investigated (Figure 5c), and 10% of all combinations giving the lowest RMSEP and RMSECV values were compared. For the original data, the highest number of combinations giving RMSEP and RMSECV values below 0.76 is achieved by the DER baseline correction method and TSN normalization method. However, RB, POL, and ME-EMSC were also located in this group of combinations. Noise added data, compared with original data, shows different trends—POL baseline correction method and TSN normalization method combinations achieve RMSEP and RMSECV lower than 1.2 the most frequently. ALS, RB, and ME-EMSC baseline correction methods also are placed below this threshold. Similarly like in the classification task, combinations with DER for noise added data seemed to be unstable in contrast to the original data.

**Figure 6:**
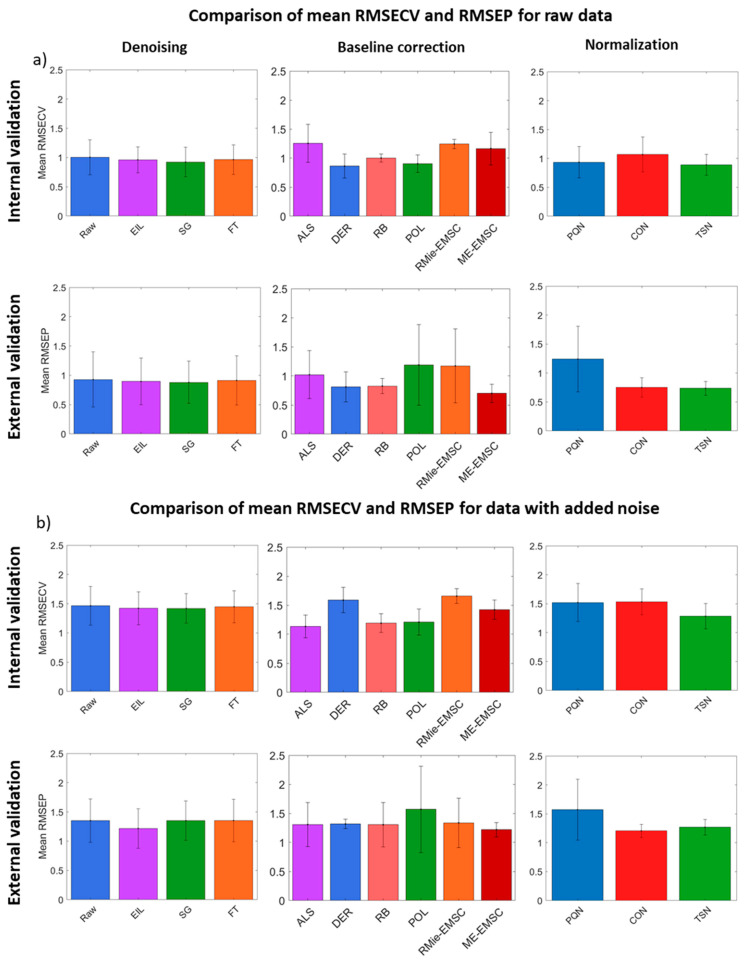
Comparison of PLSR mean RMSECV and RMSEP errors calculated for all methods on each preprocessing step (model with optimal LVs allowed by CV was chosen) for (**a**) original data and (**b**) data with added noise. The standard deviation of all models that used a given method (from the current preprocessing step) in combination with other methods (from other preprocessing steps) was marked with error bars.

For a more general overview, we also compared the mean RMSECV and RMSEP calculated for all methods on each preprocessing step with optimal LVs allowed by CV (Figure 6). A similar comparison for PLS-DA is presented in Figure S7 in Supplementary Materials. For the original data (Figure 6a), the baseline correction method gave the lowest mean RMSECV, and one of the lowest mean RMSEP is achieved with DER. The RMie-EMSC gives one of the highest mean RMSECV and RMSEP with a high standard deviation from this value. ME-EMSC gives better PLSR performance, especially on the external validation. RB and ALS methods are quite stable and give similar results in internal and external validation. Performance of the POL method is the worst in external validation in the case of mean RMSEP, but the standard deviation was highly variable, which means that this method was very sensitive for adjusting of polynomial degree, which should be done very carefully. In the case of the normalization preprocessing step, the highest mean RMSECV and its standard deviation were achieved by CON normalization, whereas TSN and PQN had a slightly better performance. Similar to classification in regression PQN method, which gives relatively good mean RMSECV values, also gave the worst result for external validation. It can be concluded that in the case of small dataset analysis, this method was the most sensitive for spectra distortions which could be passed or introduced in the preceding preprocessing steps. Therefore, after PQN normalization of such dataset, spectra become similar to each other within a given dataset (model or independent test set), and the model was unable to classify independent test samples correctly.

In the case of data with added noise (Figure 6b), it could be seen that all of the RMSECV values were significantly increased. The baseline method that suffered the most from noise was DER, which was not surprising since each order of DER adds more noise to the dataset, so it needs a high-quality input. ALS, RB, and ME-EMSC were stable and gave similar mean RMSECV and RMSEP values. Comparable to original data, POL and RMie-EMSC gave the highest standard deviation of the RMSEP. Normalization methods did not change mean RMSECV qualitatively, but PQN gave the worst mean RMSEP values, like in the case of original data. The choice of denoising method did not have a significant impact on the outcomes of PLSR, but it could be caused by an increase in general model robustness. The EIL method, which is not popular in the spectroscopy data analysis field, gave a similar result to other denoising methods. Overall, all types of analyses were highly influenced by the baseline correction method chosen, a much smaller effect from normalization, and the least from denoising. IR is a relatively high SNR technique, and denoising did not play a huge role unless the technique is pushed to its limits, e.g., two or four scans per spectrum or with high-speed imaging with QCLs [29,54].

## 4. Conclusions

The comparison of examined preprocessing methods determined the baseline correction step to have the dominant influence on data structure, and therefore, on the classification and regression results of analyzed spectra of cells. The best baseline correction method which gave the lowest RMSECV and RMSEP in original data is DER; however, this method also introduced the biggest changes in classification and regression performance for noisy data. The most stable baseline correction method in PLS-DA for noise added data was ALS, and it was also one of the most stable methods in PLSR. A new version of the physical model-based baseline correction method of ME-EMSC compared to well-known RMie-EMSC appeared to be more stable and also gave lower mean RMSECV and RMSEP. The second important preprocessing step is data normalization. For the original and noisy data, normalization to a constant and TSN gave very good results. TSN and CON are commonly used in IR spectroscopy, but sometimes they suffer from introducing additional spectral correlations. The PQN method is relatively new to the spectroscopy field and could be free of this limitation, but it has additional requirements which are not always met in small datasets. The EIL method, which is new in FT-IR spectroscopy data denoising, gave similar performance to the remaining denoising methods. However, in the case of the analyzed high SNR data with homoscedastic noise, the denoising step had little influence on improving the classification and regression performance. Therefore, further exploration of the EIL method denoising potential should be done with a lower SNR data set.

## Figures and Tables

**Table 1:** The best combination of methods gave very high internal accuracy with the smallest reasonable LVs (marked with the red circle in the left panel in Figure 3). Methods giving the best external validation values for original and raw data were marked with green color.

Denoising	Adjusted Parameter		Baseline	Adjusted Parameter		Normalization	Internal Accuracy	External Accuracy
**Original Data**								
Fourier	frame	100	Second derivative	Poly, frame	2, 27	CONSTANT	0.94	0.86
Fourier	frame	100	Second derivative	Poly, frame	2, 29	CONSTANT	0.94	0.79
Fourier	frame	100	Second derivative	Poly, frame	3, 27	CONSTANT	0.94	0.86
Fourier	frame	100	Second derivative	Poly, frame	3, 29	CONSTANT	0.94	0.79
Fourier	frame	100	Second derivative	Poly, frame	2, 23	TSN	0.94	0.86
Fourier	frame	100	Second derivative	Poly, frame	2, 25	TSN	0.94	0.86
Fourier	frame	100	Second derivative	Poly, frame	2, 27	TSN	0.94	0.86
Fourier	frame	100	Second derivative	Poly, frame	2, 29	TSN	0.94	0.86
Fourier	frame	100	Second derivative	Poly, frame	3, 23	TSN	0.94	0.86
Fourier	frame	100	Second derivative	Poly, frame	3, 25	TSN	0.94	0.86
Fourier	frame	100	Second derivative	Poly, frame	3, 27	TSN	0.94	0.86
Fourier	frame	100	Second derivative	Poly, frame	3, 29	TSN	0.94	0.86
**Noise Added Data**								
Fourier	frame	140	ALS	λ, p	6, 0.1	CONSTANT	0.91	0.86
Fourier	frame	220	ALS	λ, p	6, 0.1	CONSTANT	0.91	0.93
Eilers	λ	6	ALS	λ, p	6, 0.1	CONSTANT	0.91	0.86
SavitzkyG	Poly, frame	2, 15	ALS	λ, p	6, 0.1	CONSTANT	0.91	0.93
SavitzkyG	Poly, frame	2, 17	ALS	λ, p	6, 0.1	CONSTANT	0.91	0.86
SavitzkyG	Poly, frame	2, 19	ALS	λ, p	6, 0.1	CONSTANT	0.91	0.93
SavitzkyG	Poly, frame	2, 21	ALS	λ, p	6, 0.1	CONSTANT	0.91	0.93
SavitzkyG	Poly, frame	2, 23	ALS	λ, p	6, 0.1	CONSTANT	0.91	0.93
SavitzkyG	Poly, frame	3, 15	ALS	λ, p	6, 0.1	CONSTANT	0.91	0.93
SavitzkyG	Poly, frame	3, 17	ALS	λ, p	6, 0.1	CONSTANT	0.91	0.86
SavitzkyG	Poly, frame	3, 19	ALS	λ, p	6, 0.1	CONSTANT	0.91	0.93
SavitzkyG	Poly, frame	3, 21	ALS	λ, p	6, 0.1	CONSTANT	0.91	0.93
SavitzkyG	Poly, frame	3, 23	ALS	λ, p	6, 0.1	CONSTANT	0.91	0.93

## Data Availability

The data presented in this study are available on a reasonable request from the corresponding author.

## Supplementary Materials

The following are available online at https://www.mdpi.com/article/10.3390/cells10040953/s1, Figure S1: Spectra of cell lines marked with individual colors after CO_2_ removal for (a) original data; (b) data with added noise. Figure S2: Principal Component Analysis exploration of the data with added noise after application of the following preprocessing approach denoising → baseline correction → normalization; Figure S3: Spectra for the test set and beta coefficients for the best combination of methods which gave very high internal accuracy with the smallest reasonable LVs (marked with a red circle in the left panel in Figure S3: (a) original data and (b) noise added data; Figure S4: Spectra for the test set and beta coefficients for the worst combination of methods which gave very high internal accuracy and the worst external accuracy (marked with a green circle in the left panel in Figure 3: (a) original data and (b) noise added data; Figure S5: Internal and external accuracy values comparison for the best LVs for (a) original data and (b) noise added data, for baseline correction methods: ALS, polynomial, rubber band; Figure S6: Comparison of RMSECV and RMSEP values for the best LVs for (a) original data and (b) noise added data. Each dot on the plot presents a value for one combination; Figure S7: Comparison of PLS-DA accuracies calculated for all methods on each preprocessing step (model with optimal LVs allowed by CV was chosen) for (a) original data; (b) data with added noise.
