# Supplementary material for: The Impact of Preprocessing Methods for a Successful Prostate Cell Lines Discrimination Using Partial Least Squares Regression and Discriminant Analysis Based on Fourier Transform Infrared Imaging
Source: Cells. 2021 Apr 20;10(4):953. doi: 10.3390/cells10040953 (PMC8073124; doi:10.3390/cells10040953)
Supplement: Supplementary file 1 [file cells-10-00953-s001.zip › cells-1174509-supplementary.pdf]

Supplementary Materials

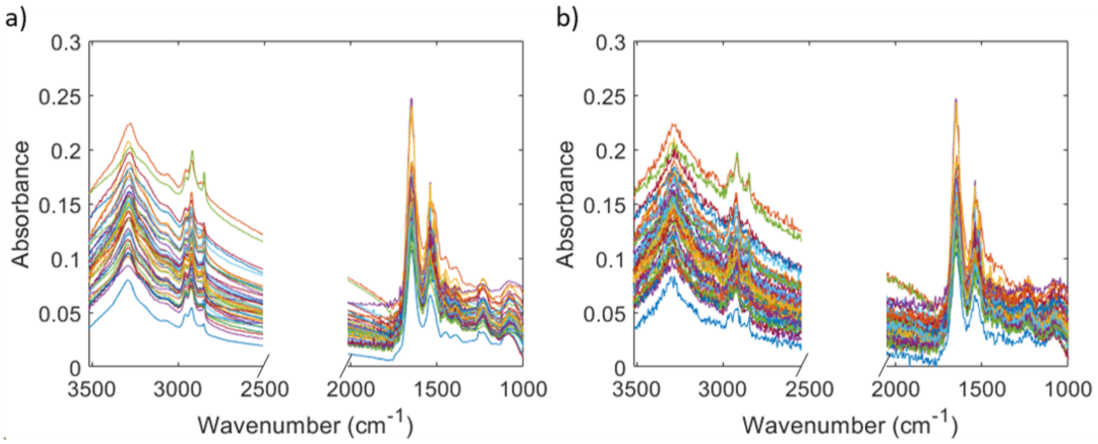

Figure S1. Spectra of cell lines marked with individual colors after CO_2 removal for a) original data; b) data with added noise.

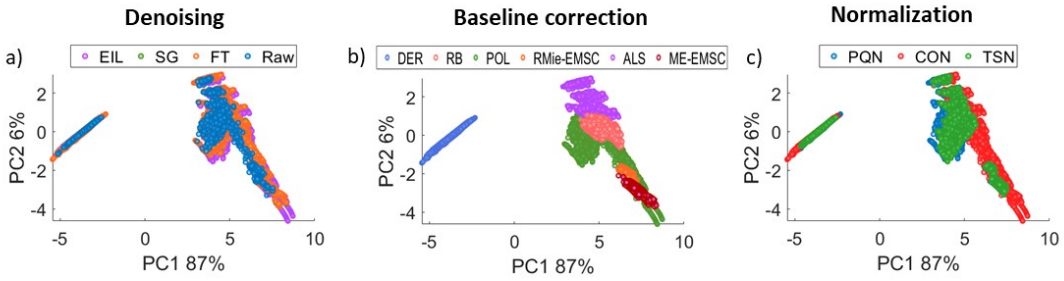

Figure S2. Principal Component Analysis exploration of the data with added noise after application of the following preprocessing approach denoising → baseline correction → normalization. Subsections a, b, and c present one PCs projection colored according to a single preprocessing type: a) Denoising, b) Baseline correction, c) Normalization. For better understanding, a set of spectra on which combinations of DER baseline correction method with other preprocessing steps were marked with a circle.

In Figure S2 it can be spotted that differences between PCA explorations are not clearly visible, they are similar to PCA results for original data. It means that for analyzed data, baseline correction has the largest impact on the data structure.

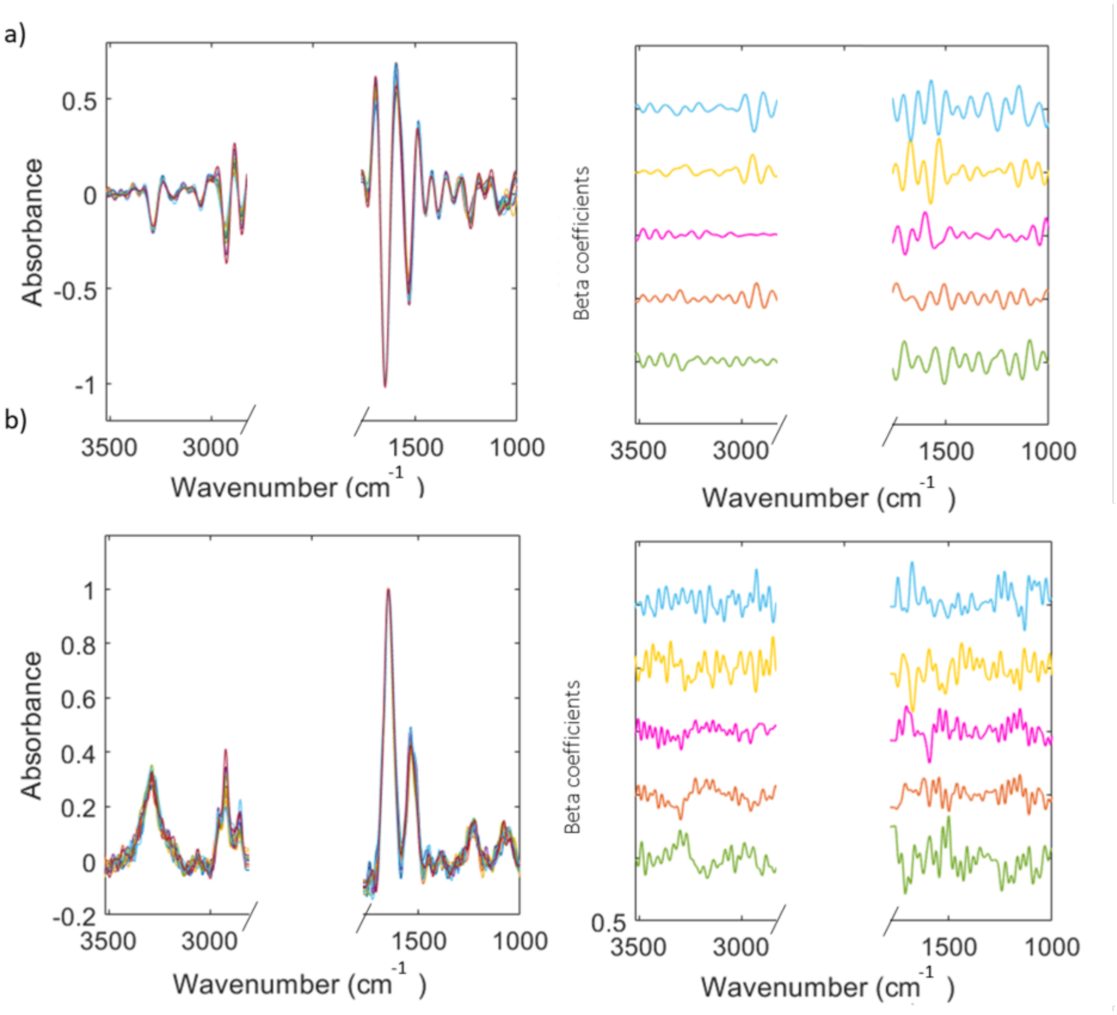

Figure S3. Spectra for the test set and beta coefficients for the best combination of methods which gave very high internal accuracy with the smallest reasonable LVs (marked with a red circle in the left panel in figure 3: a) original data, b) noise added data.

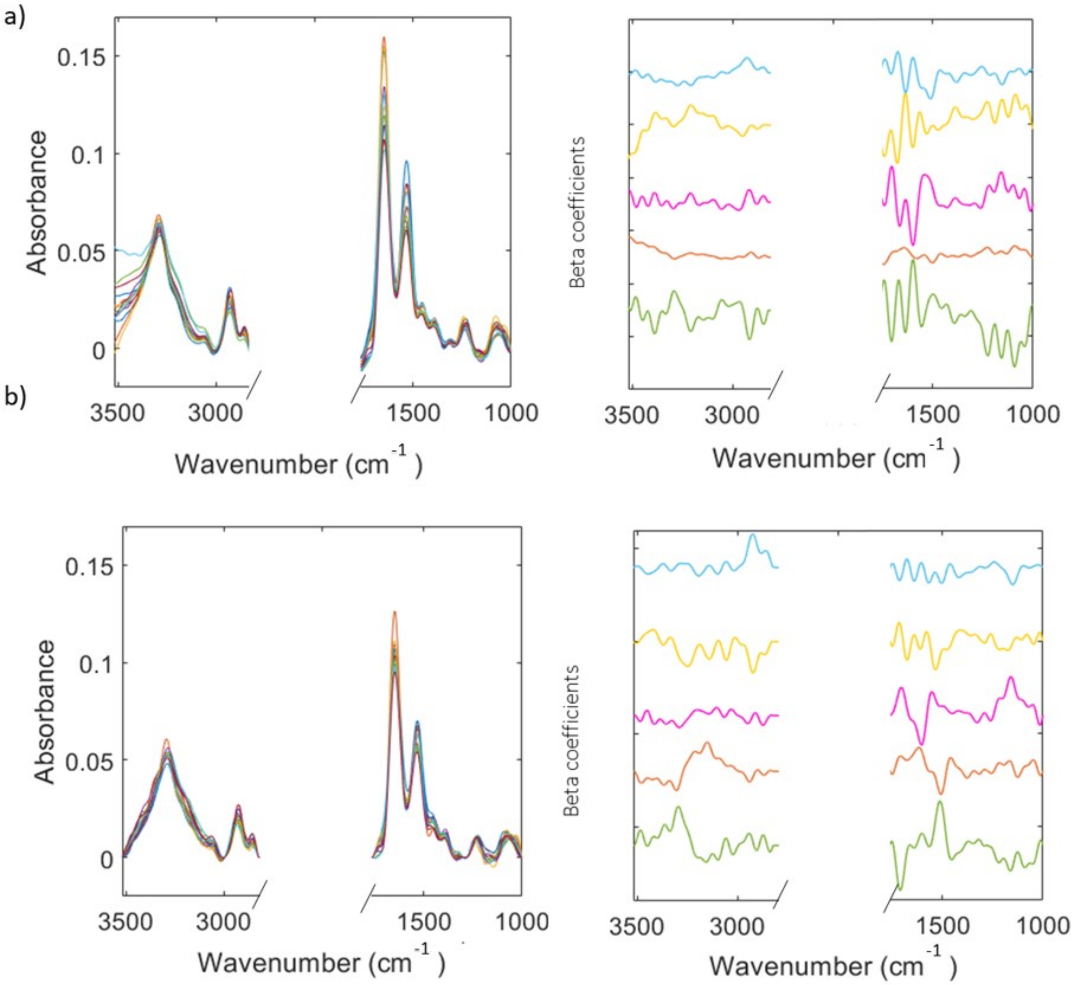

Figure S4. Spectra for the test set and beta coefficients for the worst combination of methods which gave very high internal accuracy and the worst external accuracy (marked with a green circle in the left panel in figure 3: a) original data, b) noise added data.

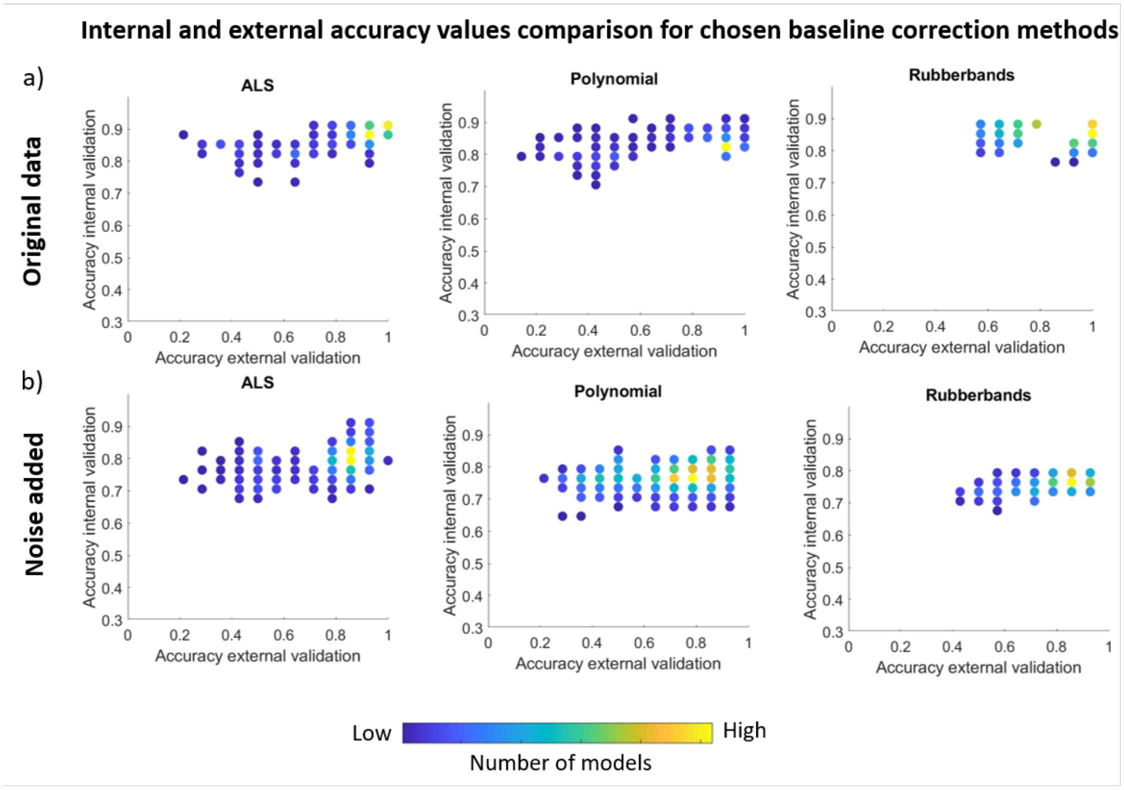

Figure S5. Internal and external accuracy values comparison for the best LVs for: a) original data and b) noise added data, for baseline correction methods: ALS, Polynomial, Rubberbands.

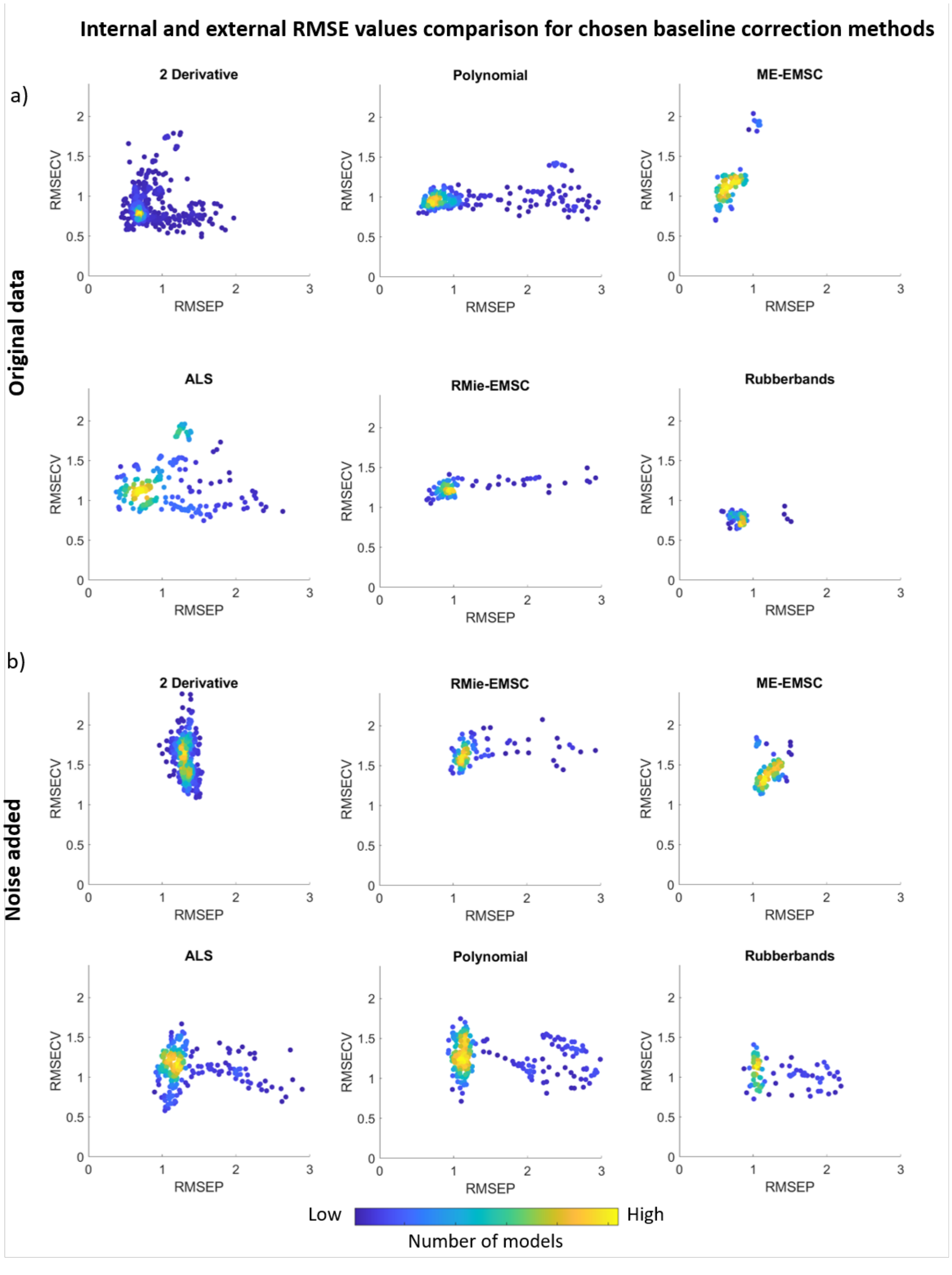

Figure S6. Comparison of RMSECV and RMSEP values for the best LVs for: a) original data, b) noise added data. Each dot on the plot presents a value for one combination.

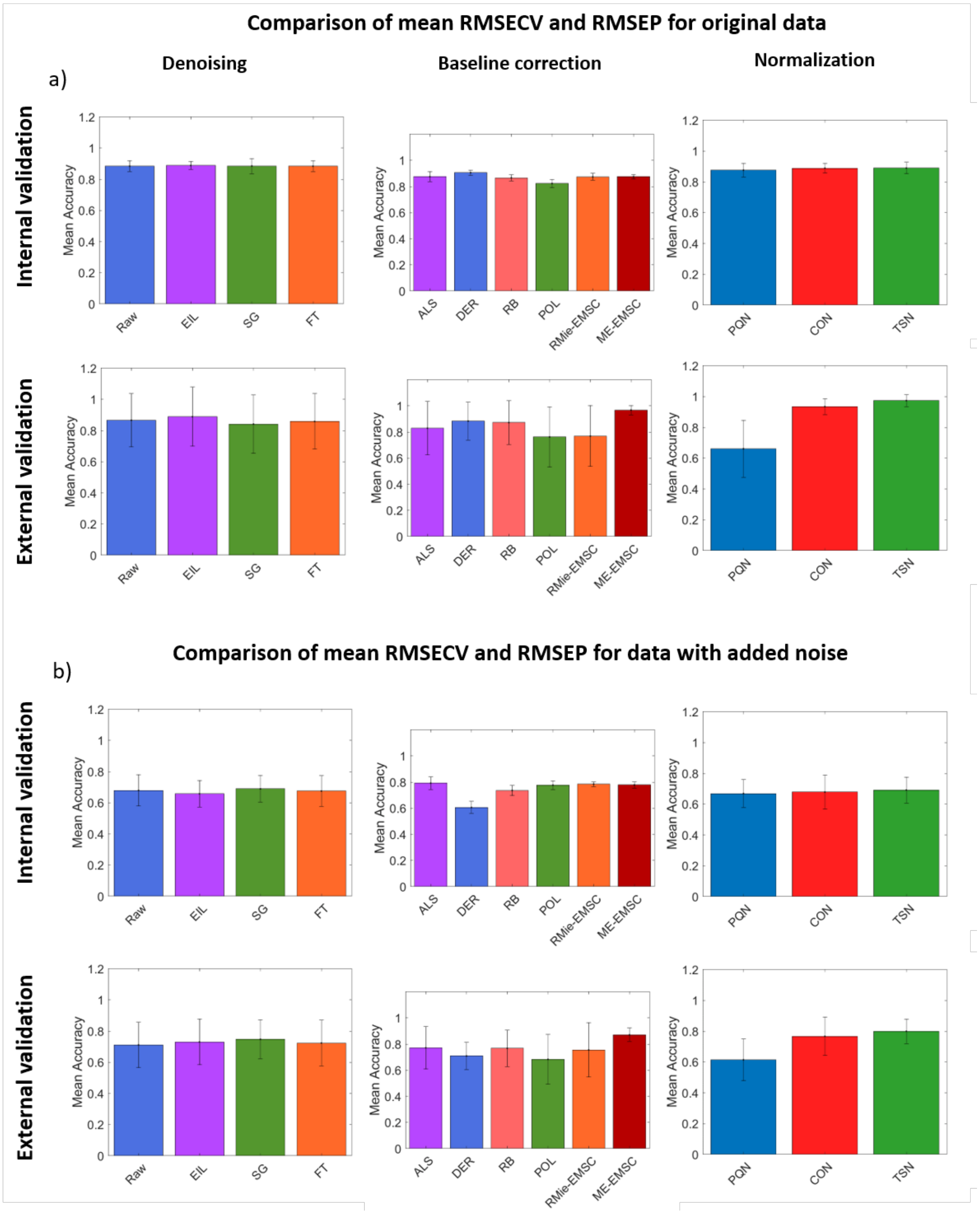

Figure S7. Comparison of PLS-DA accuracies calculated for all methods on each preprocessing step (model with optimal LVs allowed by CV was chosen) for a) original data; b) data with added noise. The standard deviation of all models that used a given method (from the current preprocessing step) in combination with other methods (from other preprocessing steps) was marked with error bars.
